# Newborn Screening for Neonatal Intrahepatic Cholestasis Caused by Citrin Deficiency and Analysis of *SLC25A13* Gene Mutations in Hefei, China

**DOI:** 10.3390/ijns12030058

**Published:** 2026-07-28

**Authors:** Qingqing Ma, Junxing Chen, Yong Huang, Yan Wang, Wangsheng Song, Hongyu Xu, Yuhui Wan, Haili Hu

**Affiliations:** 1 Department of Maternal, Child and Adolescent Health, School of Public Health, Anhui Medical University, Hefei 230022, China; 2435010227@stu.ahmu.edu.cn (Q.M.);; 2 Anhui Women and Children’s Medical Center, Hefei 230001, China

**Keywords:** neonatal intrahepatic cholestasis caused by citrin deficiency, newborn screening, *SLC25A13* gene, tandem mass spectrometry

## Abstract

Citrin deficiency (CD) is an autosomal recessive disorder and represents one of the urea cycle disorders. This study aims to analyze the detection rate, clinical features, and genetic mutation characteristics of neonatal intrahepatic cholestasis caused by citrin deficiency (NICCD) in the Hefei region of China. We conducted NICCD screening using tandem mass spectrometry (MS/MS) for infants born in Hefei City between January 2016 and December 2025. Screen-positive cases were subjected to genetic testing via next-generation sequencing (NGS), with subsequent validation by Sanger sequencing. Clinical manifestations, biochemical parameters, and genetic mutation profiles of confirmed cases were systematically analyzed. A total of 924,676 newborns underwent screening, identifying 17 cases of NICCD, yielding a detection rate of 1/54,393, with two false-negative cases identified. The most prevalent mutation site is c.852_855del (p.M285Pfs*2). Following diagnosis, health education, dietary guidance, and symptomatic treatment were administered, resulting in favorable outcomes in the majority of cases. However, one infant exhibited significant growth retardation despite early therapeutic intervention that normalized biochemical parameters. Furthermore, an infant was found to have gallstones at birth and subsequently diagnosed with a liver hemangioma at one year of age. Some patients may experience missed screenings due to delayed elevations in citrulline levels. Therefore, even for newborns with negative screening results, timely assessments of liver function, MS/MS, and genetic testing are recommended for infants experiencing prolonged jaundice. This approach enables early identification and intervention. The combination of MS/MS with genetic screening may serve as a reliable strategy to reduce false-negative results in NICCD screening.

## 1. Introduction

Citrin Deficiency (CD) is an inherited metabolic disorder, transmitted in an autosomal recessive manner, resulting from a defect in the *SLC25A13* gene which leads to a deficiency of Citrin—an aspartate–glutamate carrier protein within the mitochondrial inner membrane. It is categorized as a urea cycle disorder. Based on the age of onset and clinical features, three distinct clinical phenotypes have been classified: neonatal intrahepatic cholestasis caused by Citrin deficiency (NICCD; OMIM 605814), failure to thrive and dyslipidemia caused by Citrin deficiency (FTTDCD), and adult-onset type II citrullinemia (CTLN2; OMIM 603471) [1,2].

NICCD typically manifests within the first year of life, characterized primarily by growth retardation, jaundice, hepatomegaly, and abnormal liver function. It is often accompanied by hypoalbuminemia, coagulopathy, hemolytic anemia, hypoglycemia, and other conditions. Most clinical manifestations and laboratory abnormalities are transient, and the prognosis is generally favorable with appropriate intervention, as symptoms commonly resolve before one year of age. However, a small subset of patients may experience poor outcomes due to severe complications such as infection or cirrhosis. FTTDCD generally presents between one and two years of age. Most affected individuals exhibit a marked dietary preference for high-protein, high-fat, and low-carbohydrate intake. The primary clinical features include growth retardation and dyslipidemia [2]. A subset of patients may progress to CTLN2 over a period ranging from ten to several decades. Clinical manifestations involve recurrent episodes of hyperammonemia and associated neuropsychiatric symptoms, including seizures, behavioral abnormalities, memory impairment, disorientation, or disturbances of consciousness. In some cases, patients may succumb to severe cerebral edema [3].

Citrin deficiency is notably more prevalent in Asian populations. Epidemiological studies indicate that in Asia, the estimated prevalence of citrin deficiency, based on the carrier frequency of the *SLC25A13* gene, ranges from approximately 1/38,000 to 1/7100 [4,5,6,7]. For NICCD, early diagnosis can be achieved via tandem mass spectrometry (MS/MS) screening, which typically reveals markedly elevated citrulline levels. This metabolic biomarker has led to the inclusion of NICCD in several neonatal screening programs. Notably, a nationwide screening of approximately 7.82 million newborns in China yielded a detection rate of only 1/68,594 [8]. This relatively low detection rate may be attributed to instances of delayed-onset citrulline elevation in some patients, potentially leading to missed diagnoses. In addition, citrin deficiency has a broad phenotypic spectrum, and not all individuals with biallelic pathogenic variants in *SLC25A13* manifest NICCD in infancy. Some patients may develop other clinical phenotypes later in life, while others may remain asymptomatic or only mildly affected. Therefore, the detection rate of neonatal screening for NICCD may be lower than the theoretical genetic prevalence.

The Neonatal Disease Screening Center in Hefei, China, conducted a 10-year study (from January 2016 to December 2025), employing MS/MS to screen 924,676 newborns for congenital disorders. By analyzing data from infants diagnosed with NICCD over this period, the study aims to elucidate the detection rate, clinical characteristics, gene mutation profiles, and prognostic outcomes of NICCD in the Hefei region of China. These findings serve as a foundation for early diagnosis, treatment strategies, and genetic counseling for this condition.

## 2. Materials and Methods

### 2.1. Study Population

The study cohort comprised 924,676 newborns who underwent tandem mass spectrometry-based neonatal disease screening in Hefei, China, from January 2016 to December 2025. All sample collections were conducted in compliance with the informed consent protocol, and written informed consent was obtained from the parents or legal guardians of each participant. Our study was approved by the Ethics Committee of Anhui Women and Children’s Medical Center (Approval No: YYLL20250521-HFWJ-HYLL-04-1.0, approved on 18 June 2025), in accordance with the ethical standards of the Helsinki declaration and its later amendments or comparable ethical standards.

### 2.2. Newborn Screening for NICCD

Within 3 to 7 days after birth, three drops of heel blood are collected from the newborn after adequate feeding, applied onto a designated blood collection filter paper, and sent for testing after air-drying at room temperature. Detection was performed using a Waters TQD tandem mass spectrometer (Waters Corporation, Milford, MA, USA) employing liquid chromatography–tandem mass spectrometry (LC-MS/MS). The experimental reagents used are the underivatized multi-amino acid, carnitine and succinylacetone detection kit manufactured by PerkinElmer (Turku, Finland), which is applied to detect amino acids and acylcarnitine profiles in dried blood spots on filter paper. Citrulline (CIT) was adopted as the primary indicator, with methionine, tyrosine, arginine, and phenylalanine serving as supplementary indicators. Individuals exhibiting elevated citrulline levels were recalled as suspected cases for repeat MS/MS testing. Cases confirmed to have persistently elevated levels underwent genetic testing, along with additional laboratory assessments including hepatic and renal function, lipid profile, coagulation function, alpha-fetoprotein, and urinary organic acids.

Based on screening data from more than 300,000 newborns across multiple domestic regions using reagents from the same PerkinElmer company, as well as data collected from 20,000 newborns in the Hefei region between July and December 2015, the 0.5th and 99.5th percentiles were respectively utilized as the lower and upper limits of the analyte reference range. Accordingly, the reference interval in the present study was established as follows: Citrulline (CIT): 5.8–33.5 µmol/L; Methionine (MET): 7.92–42 µmol/L; Tyrosine (TYR): 36–229 µmol/L; Arginine (ARG): 3.5–54 µmol/L; Phenylalanine (PHE): 30–90 µmol/L; Total Bilirubin (TBIL): 3.42–20.5 µmol/L; Direct Bilirubin (DBIL): 0–7.1 µmol/L; Total Bile Acids (TBA): 0–10 µmol/L; Alanine Aminotransferase (ALT): 7–40 IU/L; Aspartate Aminotransferase (AST): 13–35 IU/L; Gamma-Glutamyl Transferase (GGT): 7–45 IU/L; Alkaline Phosphatase (ALP): 40–500 IU/L; Total Protein (TP): 64–83 g/L; Albumin (ALB): 35–52 g/L; Blood Ammonia (NH_3_): 9–33 µmol/L.

### 2.3. Genetic Analysis

Venous whole blood (2 mL) was collected from the neonates and parents using EDTA anticoagulation. Genomic DNA was extracted using a DNA isolation kit. A pre-library was constructed with a dedicated kit, followed by targeted exon capture to obtain the final library. Next-generation sequencing (NGS) was subsequently performed, covering the exonic regions and the 20-base intronic sequences flanking the exons of 85 genes, including *SLC25A13*, *ASS1*, and *ASL*. Suspected variants identified in the above genes in the proband and parents were validated by Sanger sequencing. The pathogenicity of suspected mutations detected by the above methods was evaluated according to the 2015 American College of Medical Genetics and Genomics (ACMG) guidelines. Mutations were classified into one of five categories: pathogenic, likely pathogenic, variant of uncertain significance, likely benign, or benign. Variants categorized as “pathogenic,” “likely pathogenic,” or “variant of uncertain significance” were included in this study.

### 2.4. Diagnostic Criteria [9,10]

(1) Positive findings in neonatal screening or clinical manifestations such as delayed resolution of neonatal jaundice and cholestasis in the neonatal period; (2) Elevated plasma levels of multiple amino acids, including CIT, ARG, and MET, as indicated by amino acid analysis; (3) Identification of biallelic pathogenic variants in the *SLC25A13* gene.

### 2.5. Treatment and Follow-Up

Confirmed pediatric cases were administered lactose-free formula enriched with medium-chain triglycerides (MCT), supplemented with fat-soluble vitamins, and provided with symptomatic supportive care. Upon introduction of complementary foods, dietary adjustments were guided to ensure a high-protein, high-fat, and low-carbohydrate nutritional regimen. During the initial phase of treatment, follow-up assessments were conducted monthly. After clinical symptoms and laboratory biochemical parameters substantially normalized, the follow-up interval was extended to once every three months. Monitoring during follow-up included anthropometric measurements, blood MS/MS, hepatic and renal function tests, blood ammonia levels, and cognitive development assessments.

### 2.6. Statistical Analysis

Statistical analyses were performed using SPSS version 26.0 (SPSS Inc., Chicago, IL, USA). Wilcoxon signed-rank test was employed for the analysis of paired quantitative data that did not conform to a normal distribution. A *p*-value < 0.05 was considered statistically significant.

## 3. Results

### 3.1. Screening Outcomes

**Figure 1:**
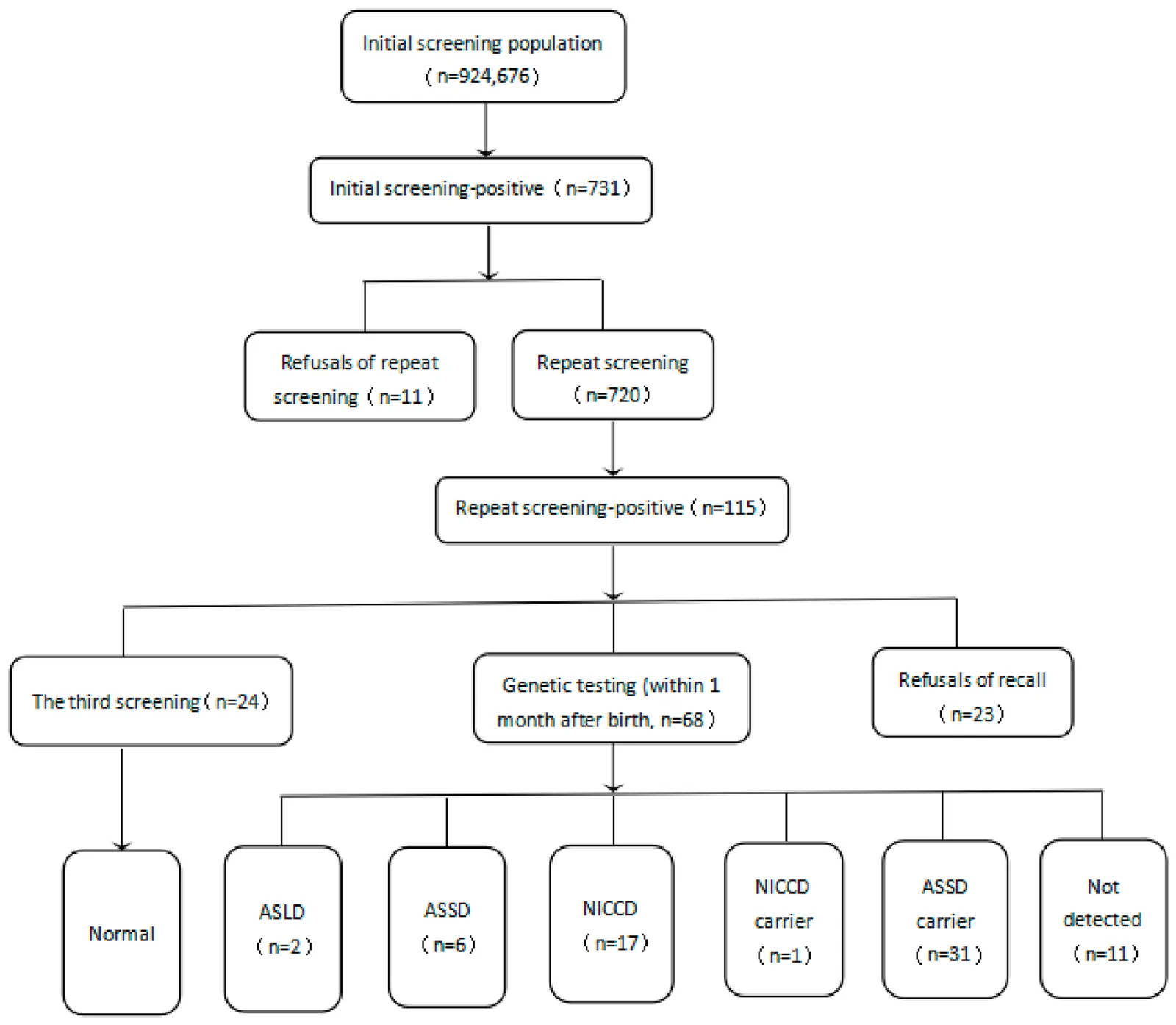
Flowchart of NICCD Screening. ASLD, argininosuccinate lyase deficiency; ASSD, argininosuccinate synthetase deficiency; NICCD, neonatal intrahepatic cholestasis caused by citrin deficiency.

A total of 924,676 newborns were screened, with 731 identified as suspected positive cases, yielding a suspected positive rate of approximately 0.079%. Among these, 11 individuals declined follow-up re-examination. The newborn screening center recalled 720 cases for re-screening, of which 115 tested positive. Of these, 23 cases refused further diagnostic recall, while 24 underwent a third-tier MS/MS test, which returned normal results. Genetic testing was performed on 68 individuals, leading to the confirmation of 17 cases of NICCD, corresponding to a detection rate of 1/54,393. In addition, we also detected 6 pediatric cases of citrullinemia type I (Figure 1). We identified 2 cases of NICCD that tested negative on tandem mass spectrometry screening but were definitively diagnosed: one involved a male twin who had a normal screening result but later developed cholestasis and was subsequently diagnosed with NICCD; the other case had a normal MS/MS screening result, but genetic screening revealed compound heterozygous variants in the *SLC25A13* gene, and the individual was later diagnosed with NICCD at another institution. Admittedly, we cannot rule out the possibility that some NICCD cases that tested negative on screening but were clinically diagnosed have not been identified in our study. A total of 34 study participants declined to undergo further examinations, which may also have an impact on the actual detection rate of NICCD in this study. In total, 19 cases were confirmed, comprising 11 males and 8 females, all of whom were born full-term. Among the confirmed cases, Case 8 and Case 9 were twins, Case 5 was one of a twin pair with a healthy female sibling, and Case 10 and Case 11 were biological sisters. Eleven of the 19 confirmed cases were small-for-gestational-age (SGA) infants (Table 1).

In the cohort, 17 neonates initially screened positive by MS/MS, presenting with hypercitrullinemia accompanied by elevations in multiple amino acids. The initial screening revealed maximum and minimum CIT levels of 527.81 μmol/L and 40 μmol/L, respectively, while the follow-up screening showed corresponding values of 732.8 μmol/L and 82.26 μmol/L. No statistically significant difference was observed in CIT levels between initial and follow-up screenings (z = 1.16, *p* = 0.246). Case 9 exhibited a normal initial screening result with a CIT level of 27.97 μmol/L. However, due to significantly elevated CIT in the initial screening of his twin brother (Case 8), which measured 162.06 μmol/L, and a follow-up CIT level of 82.26 μmol/L accompanied by increased MET and ARG, both siblings were recalled at one month of age. At that time, Case 9 had already been hospitalized at an external institution for cholestasis, with a reported bile acid level of 168.13 μmol/L. Genetic testing confirmed the diagnosis of NICCD in both individuals. Case 19 demonstrated a normal initial MS/MS screening, with a CIT level of only 15.76 μmol/L and no concomitant elevations in other amino acids. Neonatal genetic screening identified compound heterozygous variations in *SLC25A13*. Subsequent MS/MS analysis at an external institution revealed CIT levels exceeding 300 μmol/L accompanied by cholestasis (specific report unavailable), leading to a diagnosis of NICCD (Table 1).

Urinary organic acid analysis was performed on 11 pediatric patients, all of whom exhibited elevated levels of 4-hydroxyphenyllactic acid (4-HBLC). With the exception of Case 3, ten patients showed increased urinary 4-hydroxyphenylpyruvic acid (4-HBPC). Pretreatment biochemical data were collected from all 11 cases, revealing that ten patients had more than a tenfold increase in TBA levels, accompanied by significant elevations in bilirubin and GGT. In Case 6, mild increases in TBA and TBIL were observed, although DBIL was notably elevated, accounting for 46.9% of the TBIL. Ten cases exhibited mild elevations in AST, two showed mild increases in ALT, and eight had elevated ALP. TP levels were reduced in all 11 patients. Only Case 7 had ALB levels at the lower threshold of normal, while the remaining cases exhibited hypoalbuminemia. NH_3_ levels were measured in nine patients, with no significant abnormalities detected. Hepatic ultrasonography indicated hepatic steatosis in Cases 1, 3, 4, and 18, with Case 4 also presenting with cholecystolithiasis (Table 1 and Table 2).

### 3.2. Genetic Analysis of Newborns with NICCD

**Figure 2:**
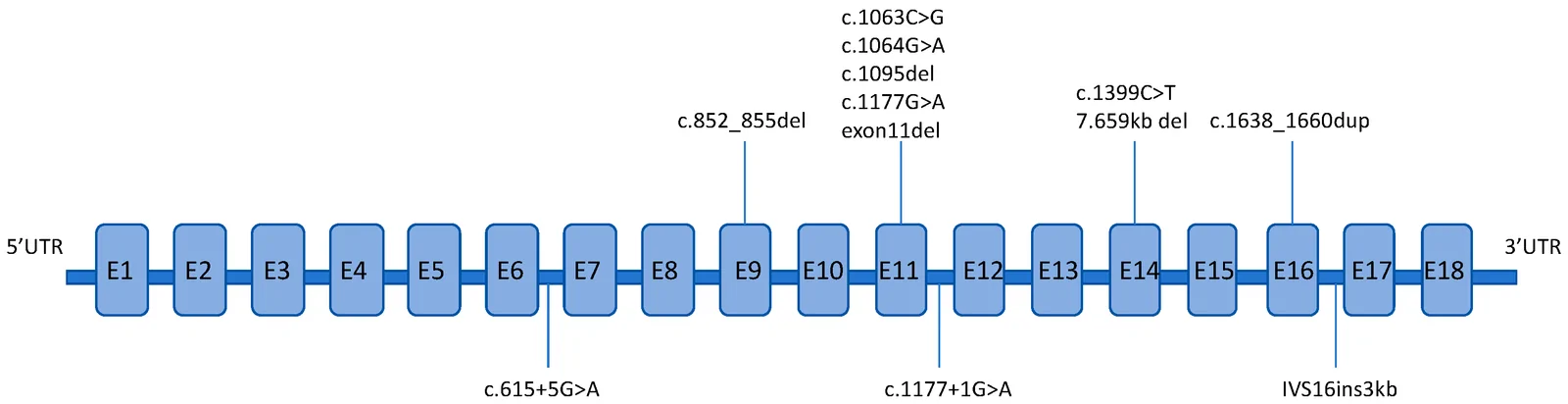
The distribution of 12 mutation types in the *SLC25A13* gene.

**Figure 3:**
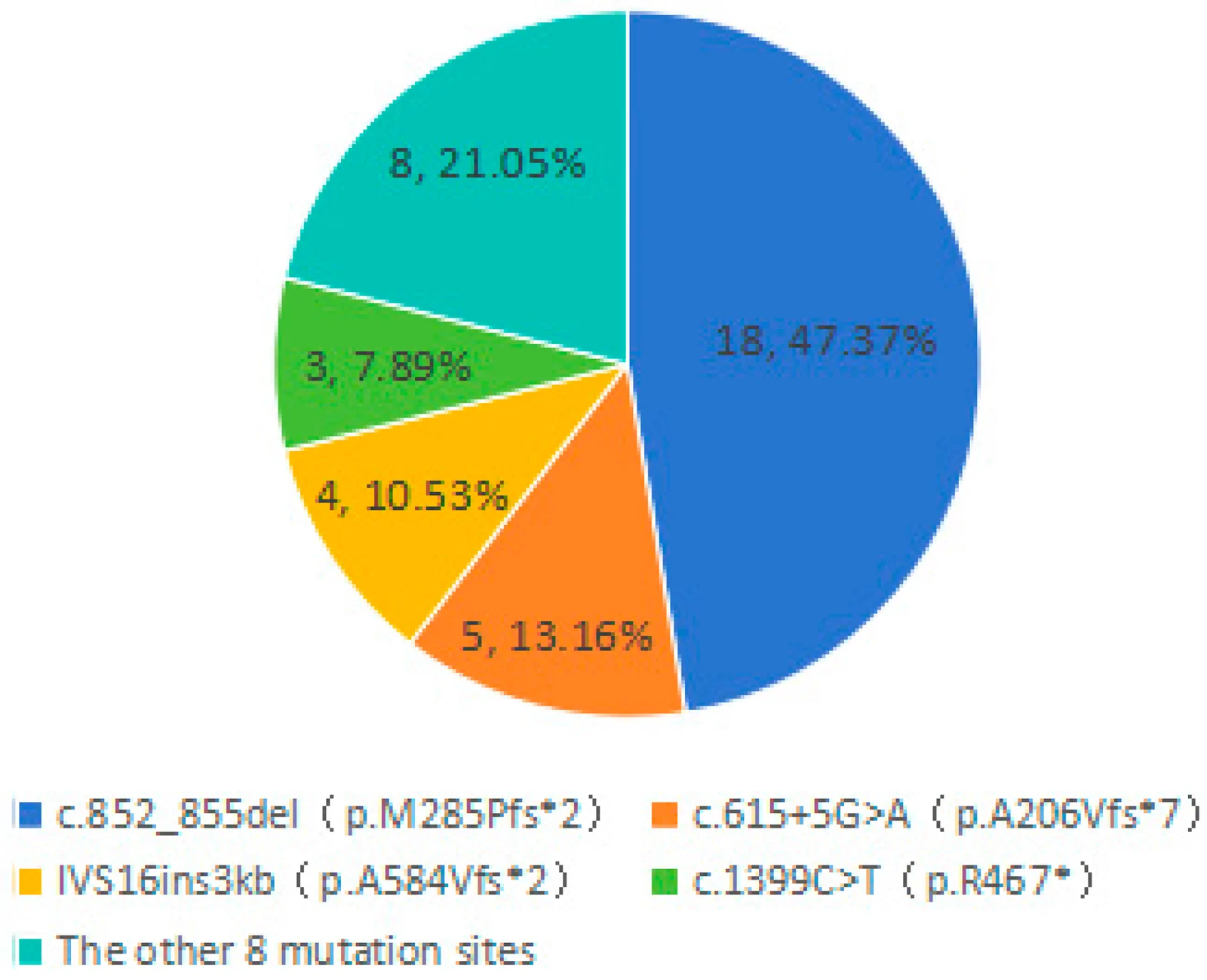
Number and percentage of mutation sites in the *SLC25A13* gene.

In 19 pediatric cases subjected to high-throughput NGS with Sanger verification, genetic analysis revealed biallelic (composite heterozygous) mutations in the *SLC25A13* gene in 13 cases. Among these, 5 cases exhibited a homozygous c.852_855del (p.M285Pfs*2) mutation, and 1 case presented a homozygous IVS16ins3kb (p.A584Vfs*2) mutation. A total of 38 mutation sites were identified, encompassing 12 different mutation types, including 4 frameshift mutations, 3 missense mutations, 2 splicing mutations, 2 deletion mutations, and 1 nonsense mutation. These variants were distributed across exons 9, 11, 14, and 16, as well as introns 6, 11, and 16 (Table 3 and Figure 2). The most frequent mutation was c.852_855del (p.M285Pfs*2), accounting for 47.37% (18/38), followed by c.615+5G>A (p.A206Vfs*7) at a mutation frequency of 13.16% (5/38) (Figure 3).

### 3.3. Treatment Outcomes and Follow-Up Results

Among the 19 confirmed pediatric cases, 15 were treated and followed up at our center. Of these, 14 were managed solely with a lactose-free formula rich in MCT, supplemented with fat-soluble vitamins. All these infants showed normalized results in blood MS/MS, TBIL, and TBA levels by 3 to 6 months of age. Case 4 was initially treated with the same MCT-rich, lactose-free formula and fat-soluble vitamin supplements for one month. By this time, TBIL and DBIL had largely returned to normal levels. However, TBA were elevated at 182.4 μmol/L, along with increased ALT (74 U/L), AST (97 U/L), and NH_3_ (63 μmol/L). Hepatic ultrasound revealed hepatic steatosis and a 5 mm × 3 mm gallstone. Treatment was subsequently augmented with ursodeoxycholic acid and compound glycyrrhizin tablets. By 4 months of age, TBA, ALT, AST, and NH_3_ levels had all normalized. A repeat ultrasound indicated persistent hepatic steatosis, with the gallstone slightly enlarged to 7 mm × 4 mm. At one year of age, ultrasound showed hepatic steatosis, a well-defined 20 mm × 13 mm hyperechoic mass in the right hepatic lobe suggestive of a hemangioma, and a gallstone measuring 9 mm × 5 mm. The patient was followed up regularly in pediatric surgery. Around 2 years of age, whole milk was introduced into the diet, while MCT-rich lactose-free formula intake was maintained at approximately 200 mL daily. The child exhibited a preference for meat, with limited consumption of vegetables and rice. At 2.5 years of age, elevated ALT (143 U/L) and AST (77 U/L) levels were noted, with other parameters remaining normal. Whole milk was discontinued, and the intake of MCT-rich lactose-free formula was increased. A follow-up examination one month later showed that liver function had returned to normal.

The 15 cases were followed up for a period ranging from 7 to 84 months. The majority of pediatric patients exhibit satisfactory growth and physical development. Case 6, at 7 months of age, presented with a height of only 61 cm (−3 SD to −2 SD) and a weight of merely 4.5 kg (<−3 SD), and was diagnosed with moderate growth retardation, severe underweight, and severe wasting. Concurrent Ages & Stages Questionnaire (ASQ) screening revealed that communication, gross motor skills, fine motor skills, and personal-social skills were below cutoff values, with problem-solving ability nearing the cutoff threshold. This infant, born small for gestational age (SGA), was delivered at 39 + 6 weeks of gestation with a birth weight of 2460 g. Following the diagnosis of NICCD at one month of age, feeding with a lactose-free, MCT-enriched formula supplemented with fat-soluble vitamins was initiated. Re-examination at two months showed normalization of MS/MS results for CIT (21.35 μmol/L), along with restoration of TBIL and DBIL to normal levels, while TBA increased to 30.61 μmol/L. Continued feeding with the lactose-free, MCT-enriched formula was advised. Due to growth retardation, the infant’s parents discontinued the prescribed lactose-free MCT formula at 5 months of age and switched to a special preterm formula. Subsequent re-evaluation by MS/MS and biochemical testing was declined thereafter.

## 4. Discussion

Citrin deficiency was initially identified in Japanese patients, with subsequent reports from other East Asian countries contributing to a growing number of documented cases in this region [3]. In 2007, the first case of European descent was reported [11], followed by published studies from various European nations, the United States, and the Middle East [12,13,14,15]. The detection rates of NICCD exhibit minor variations across different regions of China. In Zhejiang Province, located in East China, screening of 1.86 million newborns identified 29 cases, yielding a detection rate of 1/64,138, with two false negatives reported [16]. In Guangzhou, southern China, screening of 124,250 newborns detected one case, resulting in a detection rate of 1/124,250, alongside three false negatives [10]. In Heze City, northern China, screening of 296,106 newborns identified 13 cases, with a detection rate of 1/22,777 and three false negatives [17]. In this study, Hefei City is located in East China, in the central part of Anhui Province, with hilly landforms. Its ethnic composition is dominated by the Han ethnic group, which accounts for more than 99% of the total population, with ethnic minorities living in scattered communities. We screened 924,676 newborns, diagnosing 17 cases, which corresponds to a detection rate of 1/54,393, with two false negatives identified. This rate is slightly higher than those reported in neonatal screening programs in Zhejiang and Guangzhou, yet lower than the rate observed in Heze City, China.

Research conducted by Lin et al. in 2021 demonstrated specific regional variations: the *SLC25A13* gene mutation carrier rates in Guangdong Province in southern China and Shaanxi Province in northwestern China are approximately 1/51 and 1/95, respectively. Consequently, the predicted disease prevalence rates in these regions are about 1/10,053 and 1/35,865, respectively [18]. Since the launch of neonatal genetic screening in December 2023 at Hefei Neonatal Disease Screening Center, a total of 10,624 newborns have been screened by July 2025. The carrier frequency of *SLC25A13* gene mutation in this cohort is 1/69, and it is estimated that the theoretical prevalence of Citrin deficiency in Hefei area is approximately 1/19,000 (our unpublished data). The carrier frequency of *SLC25A13* gene mutation among the Chinese population shows a decreasing trend from southern to northern regions.

With the implementation of tandem mass spectrometry-based newborn screening programs, newborn screening centers across regions have successively reported false-negative cases of NICCD detected via tandem mass spectrometry screening, and some patients may be missed diagnosed due to the delayed increase in citrulline levels. A 2023 study by Chinese experts demonstrated that, compared with MS/MS screening alone, a combined approach integrating both MS/MS and genetic screening offers distinct advantages for detecting urea cycle disorders such as NICCD, which lack reliable biochemical screening markers. This integrated strategy not only shortens the time to diagnosis and improves screening efficiency, but also provides essential guidance for genetic counseling and family planning [19]. Case 19 exemplifies a false-negative MS/MS result that was subsequently diagnosed through genetic screening. With the increasing accessibility of next-generation sequencing, the adoption of molecular detection as a secondary screening method for NICCD can compensate for the limitations of tandem mass spectrometry screening. Through analysis at the genetic level, this approach provides critical confirmatory diagnostic information for suspected pediatric patients, and constitutes a key component in achieving the early detection, early diagnosis and early treatment of NICCD.

The proportion of newborns with low birth weight was approximately 57.89% (11/19). Existing studies hypothesize that the pathogenesis can be attributed to the fetus obtaining nutrients including amino acids and glucose from the mother via the placenta. However, fetuses affected by citrin deficiency (CD) lack citrin, which is the aspartate/glutamate carrier (AGC) protein. This defect prevents cytoplasmic reducing equivalents from being transported into mitochondria, leading to insufficient mitochondrial energy production in the fetus. Accordingly, CD may impair intrauterine fetal development [20]. Admittedly, whether this hypothesis is accurate remains to be verified by further research.

Consistent with previous domestic and international studies, MS/MS analysis in neonates with NICCD not only reveals elevated CIT levels but also shows increases in other amino acids such as MET, TYR, ARG, and PHE. This phenomenon may be attributed to the role of citrin protein as an AGC, which transports aspartate synthesized in mitochondria to the cytosol while simultaneously importing cytosolic glutamate and protons into the mitochondria. When gene mutations cause dysfunction of citrin protein, aspartate cannot be transported from mitochondria to the cytosol, leading to cytosolic aspartate deficiency. This subsequently inhibits argininosuccinate synthase activity, resulting in urea cycle disorders, impaired protein synthesis, and hepatocellular steatosis, ultimately elevating blood CIT levels. Concurrently, the organism may compensate by altering the metabolism of other amino acids to mitigate the dysfunction in the urea cycle and energy metabolism, leading to corresponding changes in amino acids such as MET and PHE [21]. Kido et al. conducted a retrospective analysis of newborn screening data from 96 NICCD patients across Japan and compared them with data from 13,704 newborns. They developed a multiparameter scoring system based on ARG, CIT, leucine (LEU), TYR, and the free carnitine/acylcarnitine ratio. When the total score derived from these five parameters reaches ≥4, the system demonstrates improved sensitivity and specificity for identifying NICCD cases, thereby enhancing the early detection rate of affected newborns [22].

*SLC25A13*, as the pathogenic gene for CD, is located on chromosome 7q21.3, spans approximately 200 kb with 18 exons, and encodes citrin—a protein composed of 675 amino acids. More than 100 mutation types of *SLC25A13* have been identified to date [23]. A genetic variant analysis of 458 NICCD patients from 29 provinces, municipalities, and autonomous regions across China conducted by Lin et al. revealed that the most common mutation sites in the Chinese population are c.852_855del at 58.33%, followed by IVS16ins3kb at 10.04% [24]. In contrast, the c.852_855del mutation accounts for 91.78% of cases in Vietnam, located in southern Asia [25], while its detection rate in patients from northern regions such as South Korea and Japan is only 30.3% and 25.9%, respectively [5,26], suggesting a potential north–south gradient in mutation frequency. The most common mutation in South Korea is IVS16ins3kb at 33.33%, whereas c.1177+1G>A is the second most prevalent mutation in Japan, occurring in 28.29% of cases. Among patients in the United Kingdom, c.1763G>A (p.R588Q) comprises two-thirds of mutations, while c.1173T>G (p.Y391*) and c.1465T>C (p.C489R) are more frequently observed in Caucasians [3]. The present study identifies c.852_855del as the most frequent mutation in the Hefei region of China, with a mutation frequency of 47.37% (18/38), followed by c.615+5G>A at 13.16% (5/38). The mutation profile observed in the Hefei region is largely consistent with the findings of Lin et al., with minor discrepancies likely attributable to data bias and limited sample size, which warrant further investigation in subsequent studies.

This study identified five cases (Cases 3, 8–10, and 11) with homozygous c.852_855del mutations. Among them, Cases 8 and 9 were twins, while Cases 10 and 11 were sisters. The initial CIT screening levels in these five patients ranged from 27.97 to 163.99 μmol/L. Case 9 was not recalled for further evaluation as the initial screening results for CIT and other amino acids were within normal limits. Additionally, Cases 2, 5, and 14 carried compound heterozygous c.852_855del/c.615+5G>A mutations, with initial CIT screening values of 422.4 μmol/L, 40 μmol/L, and 527.81 μmol/L, respectively. In Case 5, only a mild elevation in CIT was observed, with all other biochemical markers remaining normal. In contrast, Cases 2 and 14 exhibited not only significantly elevated CIT levels but also increased concentrations of MET, TYR, ARG, and PHE. This indicates that NICCD patients sharing identical genotypes may exhibit considerable variability in blood CIT levels. The accurate detection of NICCD in newborn screening via MS/MS is challenging due to the instability of post-natal CIT levels and the uncertain relationship between CIT concentration and genotype [27].

With the exception of Case 6, all 18 pediatric patients who returned for follow-up exhibited mild to moderate jaundice. They maintained adequate feeding and normal bowel habits. Laboratory findings indicated cholestasis, hypoalbuminemia, and elevated transaminase levels, while blood ammonia showed no significant increase. Some patients developed fatty liver in the early stages, and one case was complicated by gallstones. NICCD may present with nonspecific symptoms that overlap with hepatic and other metabolic disorders, posing challenges for early diagnosis. Although a marked elevation in plasma CIT is characteristic of this condition, genetic testing is crucial for confirming the diagnosis and differentiating it from citrullinemia type I [28].

In the follow-up of 15 children treated at our center, the majority received only lactose-free formula enriched with MCT, supplemented with fat-soluble vitamins. Blood MS/MS, TBIL, and TBA levels normalized in all patients by 3–6 months of age. In Case 4, treatment with lactose-free, MCT-enriched formula and fat-soluble vitamin supplementation alone demonstrated limited efficacy. Following the administration of choleretic and hepatoprotective therapy, biochemical parameters improved significantly. This patient was noted to have fatty liver and cholelithiasis shortly after birth, and an enhanced echogenic mass in the right hepatic lobe was detected at one year of age, with hepatic hemangioma currently considered the most probable diagnosis. Cases of NICCD patients presenting with hepatic hemangioendothelioma in infancy have been reported in Hong Kong (China) and Japan [29,30], suggesting that citrin deficiency may be associated with this type of liver tumor. Currently, there is no conclusive evidence linking hepatic hemangioma with NICCD; further research and accumulation of clinical data are necessary for clarification.

Case 6 was born as a full-term SGA infant. MS/MS and biochemical parameters of the infant had largely returned to normal by 2 months of age, with only mildly elevated TBA. The patient was diagnosed with moderate growth retardation, severe underweight, and severe wasting at 7 months of age. Additionally, an ASQ screening was performed, showing that all developmental domains scored below or near the cutoff threshold. The possibility that this pediatric patient has concomitant other diseases cannot be ruled out. Whether NICCD children will develop intellectual disability after early intervention requires follow-up observation of more clinical cases in the future. CD can disrupt the metabolism of carbohydrates, proteins, lipids, and other substances, as well as impair mitochondrial energy metabolism, with body weight potentially being more severely affected than height. A Japanese study involving 192 CD patients revealed that poor weight gain accounted for 32% of cases with growth impairment, whereas short stature (<−2 SD) was observed in 15%, indicating that inadequate weight gain constitutes a more severe complication in affected children [31].

In summary, the detection rate of NICCD identified via newborn screening in the Hefei region of China is 1/54,393, with the c.852_855del mutation presenting the highest observed frequency. Infants with suspected NICCD or neonatal cholestasis should undergo prompt liver function testing, MS/MS, and especially *SLC25A13* genetic testing, regardless of the NBS result. Genetic testing is important not only to confirm NICCD but also to aid in the differential diagnosis of other causes of neonatal cholestasis, such as biliary atresia, identify citrin deficiency before NICCD symptoms develop, and detect infants with milder or presymptomatic disease who may benefit from early follow-up and dietary guidance. The combination of MS/MS with genetic screening may represent a reliable strategy to reduce false-negative rates in NICCD screening.

## Figures and Tables

**Table 1:** The results of neonatal NICCD screening in Hefei from 2016 to 2025.

Case	Gender	Gestational Age (w)	Birth Weight (g)	Initial Screening (μmol/L)					Secondary Screening (μmol/L)					Elevated Fold-Increase in Urinary 4-HBLC	Elevated Fold-Increase in Urinary 4-HBPC
				CIT	MET	TYR	ARG	PHE	CIT	MET	TYR	ARG	PHE		
1	Male	39 + 0	2660 ^Δ^	114.39	50.32	260.24	8.4	183.13	273.35	237.61	159.89	67.55	71.93	49.99	2.39
2	Male	40 + 6	2900 ^Δ^	422.4	236	292.63	117.99	96.4	159.32	39.67	142.94	73.75	36.76	-	-
3	Male	40 + 3	3180	161.34	62.23	82.04	35.49	58.46	159.1	38.03	35.92	22.9	30.49	3.82	0.34
4	Male	39 + 3	3000	314.05	26.96	263.85	39.36	171.79	177.89	83.49	67.93	37.13	53.38	36.76	7.58
5	Female	37 + 0	2245 ^Δ^	40	28.46	61.96	40.67	40.54	105.5	25.15	54.19	42.84	40.85	85.24	35.62
6	Female	39 + 6	2460 ^Δ^	383.38	65.44	194.06	39.37	97.19	180.75	101.36	38.96	54.14	28.5	-	-
7	Female	37 + 2	2900	186.94	57.4	112.13	24.05	86.25	372.37	60.62	64.46	91.01	52.92	148.31	40
8	Male	37 + 4	2200 ^Δ^	162.06	32.3	238.68	32.66	88.41	82.26	99.57	65.31	90.14	47.41	-	-
9 *	Male	37 + 4	2050 ^Δ^	27.97	19.88	90.77	14.96	39.21	41.71 ^#^	35.65 ^#^	103.91 ^#^	40.27 ^#^	66.53 ^#^	-	-
10	Female	40 + 3	2960	163.99	23.69	414.4	8.91	112.67	421.09	83.79	291.17	42.7	107.4	-	-
11	Female	40 + 0	3060	66.9	15.16	148.13	2.05	66.56	302.78	137.85	253.05	34	104.39	62.74	39.61
12	Male	40 + 0	3350	64.76	35.48	164.98	43.04	64.33	295.1	242.38	132.9	177.31	38.42	123.95	30.5
13	Male	40 + 0	3000	77.15	28.74	250.43	19.12	64.69	732.8	169.2	337.57	138.34	130.75	-	-
14	Male	38 + 4	2600 ^Δ^	527.81	161.63	404.56	66.24	347.21	243	112.25	104.33	20.45	27.21	43.19	41.67
15	Female	40 + 5	2300 ^Δ^	157.27	20.68	208.4	6.41	135.48	188.36	29.4	55.34	23.57	43.69	31.23	16.67
16	Female	40 + 4	2650 ^Δ^	178.92	25.31	539.53	30.92	159.84	336.17	35.88	260.33	84.44	87.25	92.8	84.42
17	Male	40 + 1	2300 ^Δ^	183.94	23.05	163.53	23.68	125.07	254	16.6	67.46	28.02	60.14	4.92	16
18	Male	38 + 1	2350 ^Δ^	134.77	34.33	276.24	4.94	80.53	407.23	137.49	62.5	174.54	39.19	-	-
19 *	Female	39 + 0	3060	15.76	15.41	83.71	6.78	54.44	-	-	-	-	-	-	-

^#^ re-examination results after one month of treatment; * false-negative cases; ^Δ^ small-for-gestational-age infant; Elevation multiples represent the ratio of measured values to the upper limit of the reference range.

**Table 2:** Pre-treatment Biochemical Test Results for 11 Patients with NICCD.

Case	TBIL (μmol/L)	DBIL (μmol/L)	TBA (μmol/L)	ALT (IU/L)	AST (IU/L)	GGT (IU/L)	ALP (IU/L)	TP (g/L)	ALB (g/L)	NH_3_ (μmol/L)
1	103.6 ↑	25.8 ↑	225.06 ↑	24.3	50 ↑	509 ↑	714 ↑	43.5 ↓	28.2 ↓	-
2	138.9 ↑	31.8 ↑	208.6 ↑	28	78 ↑	176 ↑	763 ↑	47.1 ↓	31.1 ↓	57
4	99.8 ↑	15.54 ↑	133.2 ↑	24	47 ↑	217 ↑	316	43.42 ↓	27.39 ↓	50
5	155.04 ↑	23.9 ↑	181.57 ↑	28.9	70.4 ↑	563.9 ↑	825 ↑	40.3 ↓	30.6 ↓	60.3
6	29 ↑	13.6 ↑	25 ↑	13	23	40	425	41.9 ↓	26.7 ↓	14
7	114 ↑	80.6 ↑	400.6 ↑	50 ↑	98 ↑	291 ↑	1304 ↑	56.2 ↓	35.2	-
9	145.36 ↑	104.73 ↑	168.13 ↑	28.3	57.5 ↑	369.2 ↑	1673.6 ↑	39.1 ↓	27.9 ↓	30.1
11	106.5 ↑	75.6 ↑	169.8 ↑	62 ↑	129 ↑	231 ↑	833 ↑	48.4 ↓	33.7 ↓	28
15	199.4 ↑	27.2 ↑	181.4 ↑	20	56 ↑	153 ↑	663 ↑	54.3 ↓	31.2 ↓	29
16	209.7 ↑	43 ↑	158.4 ↑	33	63 ↑	244 ↑	655 ↑	37.6 ↓	27.1 ↓	43
18	129.6 ↑	25.2 ↑	139.08 ↑	26	53 ↑	325 ↑	496	35 ↓	24.7 ↓	20.4

↑, Above the upper limit of the reference range; ↓, Below the lower limit of the reference range.

**Table 3:** Genetic characteristics of newborns with NICCD.

Case	Mutation Site 1 (Paternal)					Mutation Site 2 (Maternal)				
	Location	Nucleotide Alteration	Amino Acid Alteration	Pathogenic Risk	Variant Type	Location	Nucleotide Alteration	Amino Acid Alteration	Pathogenic Risk	Variant Type
1	exon9	c.852_855del	p.M285Pfs*2	P	Frameshift	exon11	c.1177G>A	p.G393S	LP	Missense
2	intron6	c.615+5G>A	p.A206Vfs*7	P	Splicing	exon9	c.852_855del	p.M285Pfs*2	P	Frameshift
3	exon9	c.852_855del	p.M285Pfs*2	P	Frameshift	exon9	c.852_855del	p.M285Pfs*2	P	Frameshift
4	exon11	c.1095del	p.F365Lfs*43	P	Frameshift	exon11	exon11del	-	P	Deletion
5	exon9	c.852_855del	p.M285Pfs*2	P	Frameshift	intron6	c.615+5G>A	p.A206Vfs*7	P	Splicing
6	exon14	c.1399C>T	p.R467*	P	Nonsense	intron6	c.615+5G>A	p.A206Vfs*7	P	Splicing
7	intron16	IVS16ins3kb	p.A584Vfs*2	P	Frameshift	exon9	c.852_855del	p.M285Pfs*2	P	Frameshift
8	exon9	c.852_855del	p.M285Pfs*2	P	Frameshift	exon9	c.852_855del	p.M285Pfs*2	P	Frameshift
9	exon9	c.852_855del	p.M285Pfs*2	P	Frameshift	exon9	c.852_855del	p.M285Pfs*2	P	Frameshift
10	exon9	c.852_855del	p.M285Pfs*2	P	Frameshift	exon9	c.852_855del	p.M285Pfs*2	P	Frameshift
11	exon9	c.852_855del	p.M285Pfs*2	P	Frameshift	exon9	c.852_855del	p.M285Pfs*2	P	Frameshift
12	exon14	c.1399C>T	p.R467*	P	Nonsense	exon11	c.1063C>G	p.R355G	LP	Missense
13	exon9	c.852_855del	p.M285Pfs*2	P	Frameshift	exon11	c.1064G>A	p.R355Q	LP	Missense
14	exon9	c.852_855del	p.M285Pfs*2	P	Frameshift	intron6	c.615+5G>A	p.A206Vfs*7	P	Splicing
15	intron6	c.615+5G>A	p.A206Vfs*7	P	Splicing	exon16	c.1638_1660dup	p.A554Gfs*17	P	Frameshift
16	intron16	IVS16ins3kb	p.A584Vfs*2	P	Frameshift	intron16	IVS16ins3kb	p.A584Vfs*2	P	Frameshift
17	intron11	c.1177+1G>A	p.340_392del	P	Splicing	exon9	c.852_855del	p.M285Pfs*2	P	Frameshift
18	intron16	IVS16ins3kb	p.A584Vfs*2	P	Frameshift	7q21.3	7.659kb del	-	LP	Deletion
19	exon9	c.852_855del	p.M285Pfs*2	P	Frameshift	exon14	c.1399C>T	p.R467*	P	Nonsense

P, Pathogenic; LP, Likely pathogenic.

## Data Availability

The datasets generated and analyzed during the current study are not publicly available due to participant privacy concerns but are available from the corresponding authors on reasonable request.

## Abbreviations

NICCD	neonatal intrahepatic cholestasis caused by citrin deficiency
MS/MS	tandem mass spectrometry
NGS	next-generation sequencing
CD	Citrin Deficiency
FTTDCD	failure to thrive and dyslipidemia caused by Citrin deficiency
CTLN2	citrullinemia type II
MCT	medium-chain triglycerides
SGA	small for gestational age
AGC	aspartate/glutamate carrier
ASQ	Ages & Stages Questionnaire
